# Genetic evaluation of flowering, maturity time and productivity in almond (*Prunus dulcis*): heritability estimates and breeding value predictions

**DOI:** 10.1186/s12870-026-08458-1

**Published:** 2026-03-04

**Authors:** María del Mar Gómez-Abajo, Thomas M. Gradziel, Patrick J. Brown, Federico Dicenta, Pedro José Martínez-García

**Affiliations:** 1 Department of Plant Breeding, CEBAS-CSIC, PO Box 164, Espinardo, Murcia, E-30100 Spain; 2 Department of Plant Sciences, University of California, Davis, CA 95616 USA

**Keywords:** almond, breeding, flowering, genetic trends, inheritance, maturity, mixed models, productivity

## Abstract

**Background:**

Almond [*Prunus dulcis* (Mill.) D.A. Webb] is a major nut crop with high genetic complexity due to its heterozygosity and self-incompatible origin. In this study, genetic parameters for flowering, maturity and productivity, three key complex traits in almond breeding, were estimated using classical methods (midparent-offspring regression and between/within family variance components) as well as a Bayesian linear mixed model.

**Results:**

A comprehensive dataset from the CEBAS-CSIC Almond Breeding Program (CC-ABP), comprising over 17,500 individuals and more than 30 years of historical phenotypic records, was used to generate the first complete pedigree and evaluate trait inheritance. Narrow-sense and broad-sense heritability estimates were obtained, showing substantial variation across traits and methods, with the highest values derived from classical approaches. Bayesian mixed models, implemented via the MCMCglmm R package, allowed for the estimation of breeding values (EBVs), repeatability, and variance components under unbalanced data conditions. Repeatability estimates ranged from 0.15 to 0.56. EBVs were calculated for all individuals, including those without phenotypic records, enabling the construction of trait-specific rankings for early or late flowering, maturity and productivity. Reliability values were used to refine these rankings, improving the accuracy of parental selection. Genetic trends based on EBVs revealed changes in breeding objectives over time and highlighted the limited genetic progress achieved through phenotypic selection alone.

**Conclusions:**

The integration of the complete pedigree, EBVs, and trait rankings offers a robust framework to optimize crossing strategies. This work lays the groundwork for incorporating genomic selection into almond breeding efforts, improving selection efficiency for traits of agronomic importance.

**Supplementary Information:**

The online version contains supplementary material available at 10.1186/s12870-026-08458-1.

## Introduction

 The almond (*Prunus dulcis* (Mill.) D.A. Webb), belonging to the subgenus *Amygdalus* within the genus *Prunus* (Rosaceae), is believed to have originated in Central Asia, spreading to Western Asia and the Mediterranean through trade routes [1]. Its cultivation is concentrated in regions with Mediterranean-type climates, with the United States (California), Australia, and Spain as the main producers. Almonds are classified botanically as drupes. However, unlike other *Prunus* fruits such as peach or cherry, the edible part is the seed [2]. Almond has been predominantly self-incompatible and requires cross-pollination to achieve high yields.

In this sense, self-compatibility has been one of the main goals of almond breeding [3–7]. Since the 20th century, several breeding programs have emerged, notably in Spain (CEBAS-CSIC, IRTA, CITA), the United States (UC Davis, USDA), and Australia (University of Adelaide), leading to the release of numerous cultivars among them ‘Penta’, ‘Makako’, ‘Florida’ and ‘Alaska’ (CEBAS-CSIC), ‘Constantí’, ‘Vairo’ and ‘Marinada’ (IRTA), ‘Belona’, ‘Soleta’ and ‘Mardía’ (CITA), ‘Sweetheart’ and ‘Winters’ (UC Davis), ‘Yorizane’ (USDA), ‘Independence’ (Zaiger Genetics) and ‘Capella’, ‘Carina’ and ‘Maxima’ (University of Adelaide).

In Spain, another key breeding objective has been the development of late-flowering cultivars to reduce frost damage [8, 9]. As a result, flowering time has become a critical trait. This trait is determined by chilling and heat requirements of each cultivar, and it is highly dependent on environmental conditions [10, 11]. More recently, under the current climate change, early flowering, with low chilling requirements, cultivars are gaining interest for their adaptability to new warm production areas [12, 13].

Other traits of interest for breeders include flower density, maturity time, and productivity [14–19]. Flower density usually correlates with yield and could be considered a reliable productivity indicator. Early maturity is advantageous in warmer areas to avoid heat stress of summer and in colder areas to reduce the risk of fungal infection and facilitate post-harvest handling during the wet autumn [20–22]. Productivity is regarded as the most relevant trait, since plantation profitability depends on the balance between yield, kernel price and production costs.

These traits are considered complex and polygenic, with high environmental sensitivity. Thus, understanding their genetic architecture is essential to develop efficient breeding strategies. Traditional methods such as mass selection and hybridization have introduced variability [2, 23–25], but phenotypic selection alone may be inefficient due to environmental influence and low heritability of some traits.

The integration of molecular markers has enabled early selection (marker-assisted selection (MAS)), identification of cultivars and determination of genetic variability [2, 26–29] and QTL mapping [30–35]. High-throughput tools like SNP arrays [36] and long-read sequencing have enhanced genome resolution and epigenetic analysis [37]. These advances pave the way for genomic selection, which has already improved efficiency in species like eucalyptus, apple, and apricot [38–40].

In the meantime, pedigree-based prediction offers a robust and cost-effective strategy to estimate breeding values (BVs), especially for long-generation crops [41, 42]. Based on the infinitesimal additive model [43], this approach allows the calculation of Best Linear Unbiased Predictions (BLUPs) to predict genetic merit. The breeding value, defined as twice the deviation of an offspring’s mean phenotype from the population mean under random mating [44], reflects the additive genetic potential passed to progeny. Its reliability, expressed as the squared correlation between true and estimated BVs, depends on the amount and quality of pedigree and phenotypic data [45, 46]. Higher reliability leads to more accurate selection and greater genetic gain per unit time. No previous studies applied this approach to almond, although it was proven successful in other species [47, 48]. Additionally, the genetic trend, defined as the change in average BVs over time, is a useful parameter to assess breeding progress and predict future gain [49].

This study aimed to estimate breeding values, their reliability, and the genetic parameters of heritability and repeatability for four traits treated as continuous (initial, full, and final flowering time, and maturity time), and two traits treated as categorical (flower density and productivity) in almond. Repeated measures over multiple years were used to estimate repeatability. Genetic trends were analyzed to assess the evolution of the CC-ABP population. Rankings were established based on EBVs and their reliability to improve selection accuracy.

## Materials and methods

### Plant material

The breeding populations consisted of 17,581 almond trees from the CC-ABP, and they were grown in the experimental field of CEBAS-CSIC in Santomera, Murcia (SE of Spain). Out of the total, 17,529 individuals were progeny obtained from a wide range of crosses. The remaining 52 progenitors were traditional cultivars (with different origins), relative species of almond (such as *Prunus webbii*, *Prunus scoparia* or *Prunus orientalis*) and Spanish ecotypes (such as ITAP-1).

### Phenotypic traits

Once the flowering or maturation of the population began, the expert observer visited the orchards weekly to visually record these traits. Initial, full and final flowering times were scored in Julian Days, when 5%, 50% and 95% flowers of a tree were open, respectively. Full flowering time is the most common trait used to determine the flowering time of a cultivar. The maturity time was also established in Julian Days when the mesocarp was open in 95% of the fruits on the tree. Flower density was scored visually using an ordinal scale from 0 (no flowers) to 5 (very high density of flowers). Similarly, productivity was scored visually using an ordinal scale from 0 (no production) to 5 (very high production). The value 3 corresponds to a suitable productivity.

### Pedigree analysis

For the construction of the pedigree and calculation of the number of founders, the Python program PyPedal [50] was used. Inbreeding coefficients, an ordered pedigree and relationship matrix (*A*) were obtained using the R package pedigreemm [51].

### Statistical analysis

A complete descriptive statistical analysis was performed to summarize and explain the data distribution (e.g., minimum, maximum, average values). This also allowed us to identify phenotypic outliers, eliminating those that could affect the consistency of the data. Broad-sense (H²) and narrow-sense heritability (h²) were estimated for all traits. H² represents the proportion of phenotypic variance due to all genetic effects, whereas h² considers only additive genetic variance [52]. Narrow-sense heritability (h^2^) was estimated using the traditional method of midparent-offspring regression [44]. The regression model can be expressed as *Y*_*i*_ *= β*_*0*_ *+ β*_*1*_*X*_*i*_*+e*_*i*_, where *β*_*0*_ represent the intercept of the regression, *Y*_*i*_ and *X*_*i*_ represent the phenotypic means of offspring and parents in family *i*, respectively, and *e*_*i*_ is the residual error. The regression coefficient (*β*_*1*_) provides a direct estimate of h^2^ in a narrow sense. The analyses utilized simple phenotypic means of parents and offspring; although year-adjusted means were also examined to assess environmental effects, the primary heritability estimates presented are based on the unadjusted means that provides more representative estimates of the total genetic variation in such a highly unbalanced dataset. This avoids bias from a uniform correction that may not adequately capture family-specific environmental variation captured within the year effect. To address unequal family sizes, weights were applied to the regression models according to Lynch & Walsh [53]. Following this approach, the weights (*ω*) were calculated based on the family size (*n*) and the intraclass correlation (ρ) as *ω* = *n* /1+(*n*-1)*ρ*. The intraclass correlation was estimated by fitting a linear mixed model with family as a random effect to partition the variance into between-family (σ²_F_) and within-family (σ²_e_) components, where *ρ* = σ²_F_/ (σ²_F_ + σ²_e_). The second method involved partitioning variance components between and within families, following the approach described by Kearsey [54]. In this framework, heritability was approximated (H^2^ = 2σ²_F_/σ²_F_+σ²_e_), representing an estimate of broad-sense heritability.

In contrast to traditional methods, a third approach was employed to estimate heritability and to partition genetic variance components for the target traits, following the framework proposed by Martínez-García et al. [47]. For traits following a normal distribution, a linear mixed model was fitted assuming a Gaussian distribution. In contrast, for non-normally distributed traits, such as flower density and productivity, a threshold model was used. This model relates the observed categorical data to an underlying multivariate normal distribution via a probit link function [55–57]. In this case, the residual variance was fixed at 1.0, as is standard for categorical traits. Additionally, the repeated phenotypic records typical in almond breeding enabled the inclusion of both genetic effects and permanent environmental effects in the model. This structure accounts for the repeated measurements taken on the same individuals across years. The permanent environmental effect represents non-genetic variation consistent across repeated measurements of the same individual; it captures tree-specific conditions, such as micro-environmental differences or local management. The separation of these effects was possible through the use of the complete pedigree dataset, encompassing 17,581 individuals from the breeding program.

The linear model used was defined as y_ijklm_ = µ + age_i_ + year_j_ + plot_l_ + *a*_kl_ + pe_kl_ + *e*_ijklm_, where *y*_ijklm_ was the m-th observation on the k-th tree, in the l-th plot, during the j-th year, and from the i-th age class. The model includes the overall mean (µ), random effects of age class (age_i_), year (year_j_), and plot (plot_l_), as well as the additive genetic effect (*a*_kl_), the permanent environmental effect (pe_kl_), and the residual error (e_ijklm_). All explanatory variables were treated as random with weakly informative priors due to the large number of levels. The additive genetic effects, *a*_kl_, were assumed to follow a multivariate normal distribution *a*_kl_ ~ *N*(0, *A*σ^2^_a_), where *A* is the numerator relationship matrix for 17,581 trees, obtained using the *getA()* function from the pedigreemm R package. Similarly, permanent environmental effects and residuals were assumed to be independent and normally distributed pe_kl_ ~ *N*(0, *I*σ^2^_pe_) and e_ijklm_ ~ *N*(0, *I*σ^2^_e_). Age, year, and plot were modelled as independent random effects with variances σ²_year_, σ²_age_, and σ²_plot_, respectively. The main goal was to estimate µ, predict the random effects, and calculate the variances σ²_a_, σ²_pe_ and σ²_e_. These estimates allowed for the calculation of narrow-sense heritability (h^2^ = σ²_a_/(σ²_a_+σ²_pe_+σ²_e_)) and repeatability (r = (σ²ₐ+σ²ₚₑ) / (σ²ₐ+σ²ₚₑ+σ²ₑ)). Notably, variances attributed to non-genetic factors, such as year, plot, and age, were excluded from the denominator when calculating heritability and repeatability. Consequently, the estimates of these genetic parameters were considered within specific year, plot, and age groups.

Bayesian inference was performed using the MCMCglmm package in R [58], following the methodology of Martínez-García et al. [47]. Fixed effects were assigned diffuse normal priors, and random effects followed multivariate normal distributions. Variance components were estimated using inverse-Wishart priors. Each MCMC chain ran for 500,000 iterations with a 50,000 burn-in and a thinning interval of 200, yielding 2,250 posterior samples. Model convergence was assessed using trace plots, autocorrelation diagnostics, and posterior density inspection [59]. The high posterior density (HPD) intervals provided measures of precision for the estimates. Narrower HPD intervals indicated higher confidence in the posterior mode estimates.

In addition, Pearson’s correlations were calculated among traits and inbreeding coefficients were estimated using the R package pedigreemm. Finally, genetic trends were estimated using mean de-regressed BLUPs (drBLUPs) instead of average EBVs. The de-regressed BLUP (drBLUP) of each individual was calculated as drBLUP = BLUP/r_i_, where r_i_ is the reliability of the estimated breeding value. This approach reduces bias caused by individuals with limited information whose BLUPs tend to regress toward the population mean. To further account for the highly unbalanced structure of the population, genetic trends were estimated following the weighted regression methodology proposed by Garrick et al. [60]. A weighted linear regression of individual drBLUPs on year was fitted, where weights account for the number of individuals, their reliabilities, and the trait heritability. Individual weights were calculated as: ω_i_ = (1 – h^2^) / ([c + (1 − r_i_^2^) / r_i_^2^] × h^2^). Where *h*^*2*^ is the trait heritability, *r*_*i*_^*2*^ is the reliability of individual *i*, and *c* is a tuning parameter controlling the influence of individuals with low reliability. Following recommendations for breeding populations, a value of *c = 0.5* was used to balance confidence in the data while preventing individuals with extreme values and low information from disproportionately affecting the trend. Yearly weights were obtained by summing individual weights within each year, ensuring that years with more and/or more reliable individuals contributed more strongly to the estimation of the genetic trend. This results in a slope that reflects the genetic trajectory of the core breeding population.

## Results

### Data overview

Historical phenotypic data from the CC-ABP population, covering over 30 years of evaluation, were used to analyze the selected traits. The dataset included individuals with varying numbers of annual records, from 1,777 individuals evaluated in a single year to cultivars such as ‘Marcona’, ‘Ferragnès’, and ‘Desmayo Largueta’, which had records spanning over 20 years. The cultivar with the most extensive data was ‘Marcona’, with 75 observations across 22 years. On average, each individual had 2.45 records, with 1,711 individuals represented by a single observation. The average age of individuals in the breeding population was 3.71 years, with ‘Achaak’ and ‘Desmayo Largueta’ being the oldest at 33 years.

The number of families, individuals, and years varied across traits (Table 1). Flower density and full flowering time were the most consistently recorded, each with 30 years of data. Flower density had the widest representation, with 8,088 individuals, 344 families and 19,729 total data.

**Table 1:** Number of total data, number of individuals and families with data and number of years of study for each trait

	Total data	Individuals	Families	Years
Initial flowering time	6,554	2,636	77	10
Full flowering time	16,381	7,382	338	30
Final flowering time	6,520	2,616	73	9
Maturity time	8,524	4,974	306	28
Flower density	19,729	8,088	344	30
Productivity	15,097	6,701	317	29

For full flowering time, maturity time, flower density and productivity the family with the largest number of data was ‘R1000’ × ‘Desmayo Largueta’. In contrast, for initial and final flowering time, the most extensively recorded family was ‘Ramillete’ × ‘Mono’, while ‘Ramillete’ × ‘Tuono’ had the highest number of individual trees observed (Table 2).

**Table 2:** Top five families with the highest number of data recorded, and the number of individuals, for each trait

Family	Data	Individuals
**Initial flowering time**		
Ramillete x Mono	302	109
Ramillete x Tuono	282	125
Tuono x Ferragnès	269	116
Atocha x Genco	226	93
Primorskii x Garrigues	220	78
**Full flowering time**		
R1000 x Desmayo Largueta	982	304
Desmayo Largueta x R1000	607	209
Ramillete x Mono	302	109
Tuono x Ferragnès	283	116
Ramillete x Tuono	282	125
**Final flowering time**		
Ramillete x Mono	302	109
Ramillete x Tuono	282	125
Tuono x Ferragnès	268	116
Atocha x Genco	226	93
Primorskii x Garrigues	220	78
**Maturity time**		
R1000 x Desmayo Largueta	422	228
Ramillete x Tuono	207	124
Ramillete x Mono	199	108
S5133 x Antoñeta	178	74
Primorskii x Garrigues	147	75
**Flower density**		
R1000 x Desmayo Largueta	2,007	616
Desmayo_Largueta x R1000	617	209
Ramillete x Tuono	375	125
Tuono x Ferragnès	361	116
Ramillete x Mono	327	109
**Productivity**		
R1000 x Desmayo Largueta	590	235
Ramillete x Tuono	375	125
Tuono x Ferragnès	345	115
Ramillete x Mono	325	109
Antoñeta x Penta	282	160

**Fig. 1:**
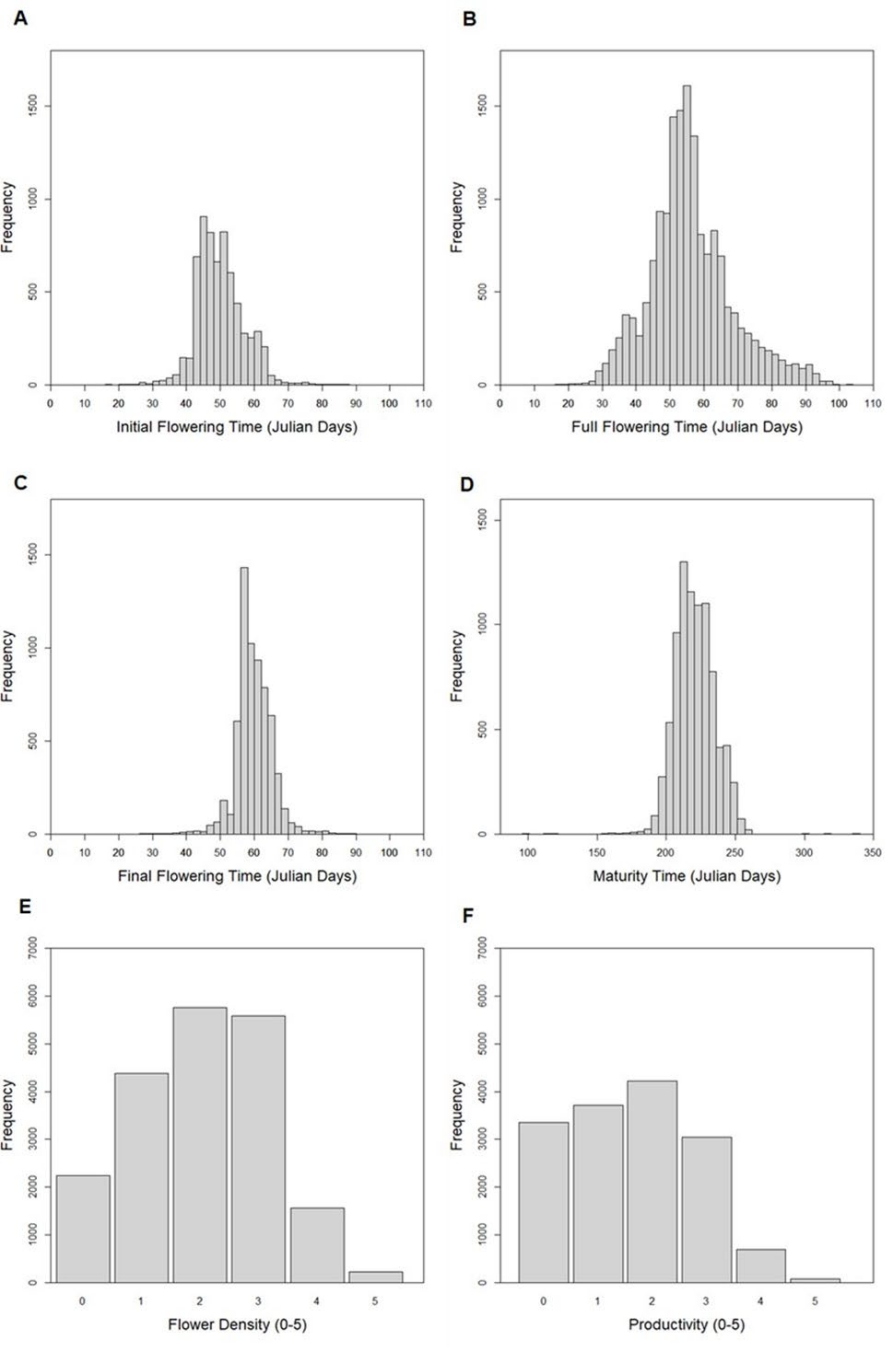
Frequency histograms of initial (**A**), full (**B**), and final (**C**) flowering times, and maturity time (**D**), all expressed in Julian days. (**E**) Frequency histogram of flower density and (**F**) productivity, both scored on a scale from 0 (no flowers or no production) to 5 (very high density or production)

Trait distribution is shown in Fig. 1. All traits displayed an approximately normal distribution, while flower density and productivity, recorded as ordinal traits, showed a roughly similar pattern, despite their ordinal score. For the breeding population, the average (considering individuals with at least two years of data) Julian day values were: 50 for initial flowering, 57 for full flowering, 60 for final flowering, and 221 for maturity time. The average flower density score was 2.02 and the average productivity score was 1.62.

Among cultivars and selections, ‘Desmayo Largueta’ and ‘Marcona’ had the most extensive records for flowering traits, with up to 23 years of data for full flowering time. For maturity time and productivity, the advanced selection D00_078 stood out with 15 years of data. In terms of phenological extremes, ‘Desmayo Largueta’ showed the earliest average initial flowering (23 Julian days), while ‘Tardona’ was the latest (82 Julian days). For full flowering, M10_006 was the earliest (25 Julian days) and D09_305 the latest (93 Julian days). For final flowering, ‘Desmayo Largueta’ again flowered earliest (37 Julian days), and ‘R1000’ latest (85 Julian days). In maturity time, D17_235 was the earliest individual with an average of 169 Julian days, while D04_341 was the latest, at 276 Julian days. Regarding flower density, score 2 was the most frequent across the population. A total of 223 individuals achieved the maximum score of 5, while 2,236 scored 0 at least once. The highest average flower density was observed in D01_631 (4.75), whereas 97 individuals maintained an average score of 0. For productivity, 2 was the most repeated score. There were 73 individuals who reached the maximum score of 5, while 2,708 exhibited a productivity score of 0 in at least one observation. The highest average productivity was 5, reached by 8 individuals, and the lowest was 1, reached by 291 individuals.

## Correlations between traits

**Fig. 2:**
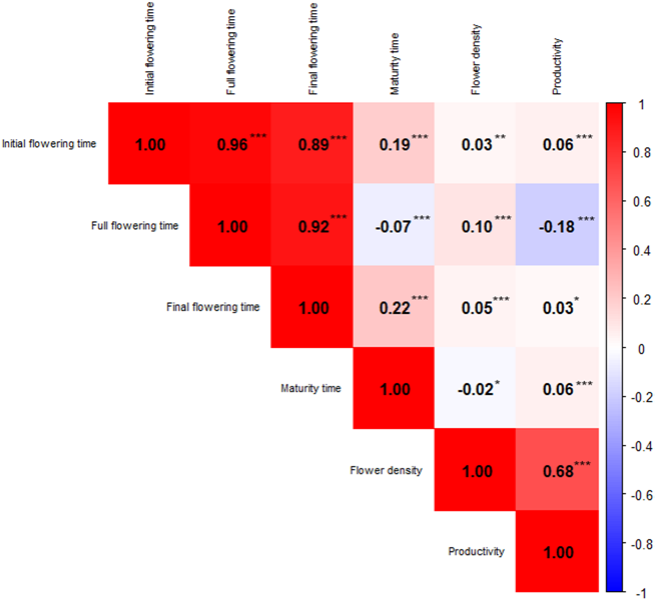
Pearson correlation matrix between all the studied traits by corrplot analysis. Asterisks indicate significance levels: *p* < 0.05 (*), *p* < 0.01 (**), and *p* < 0.001 (***)

All correlation coefficients were statistically significant (Fig. 2). In general, the correlations between flowering time traits were very high. The strongest correlation was found between initial and full flowering time (*r* = 0.96), while the weakest, although still high, was between initial and final flowering time (*r* = 0.89). In contrast, flower density showed the lowest correlations with the rest of the traits except for productivity, with which it presents a high correlation of 0.68. The rest of the correlations involving flower density showed coefficients close to zero. Maturity time presented weak correlations with flowering date traits, showing the highest with initial flowering time (*r* = 0.19) and final flowering time (*r* = 0.22), which are still very low. Correlations of maturity time with flower density and productivity were negligible. Productivity showed correlations close to 0 for the flowering date traits except for full flowering (*r* = -0.18).

## Pedigree

**Fig. 3:**
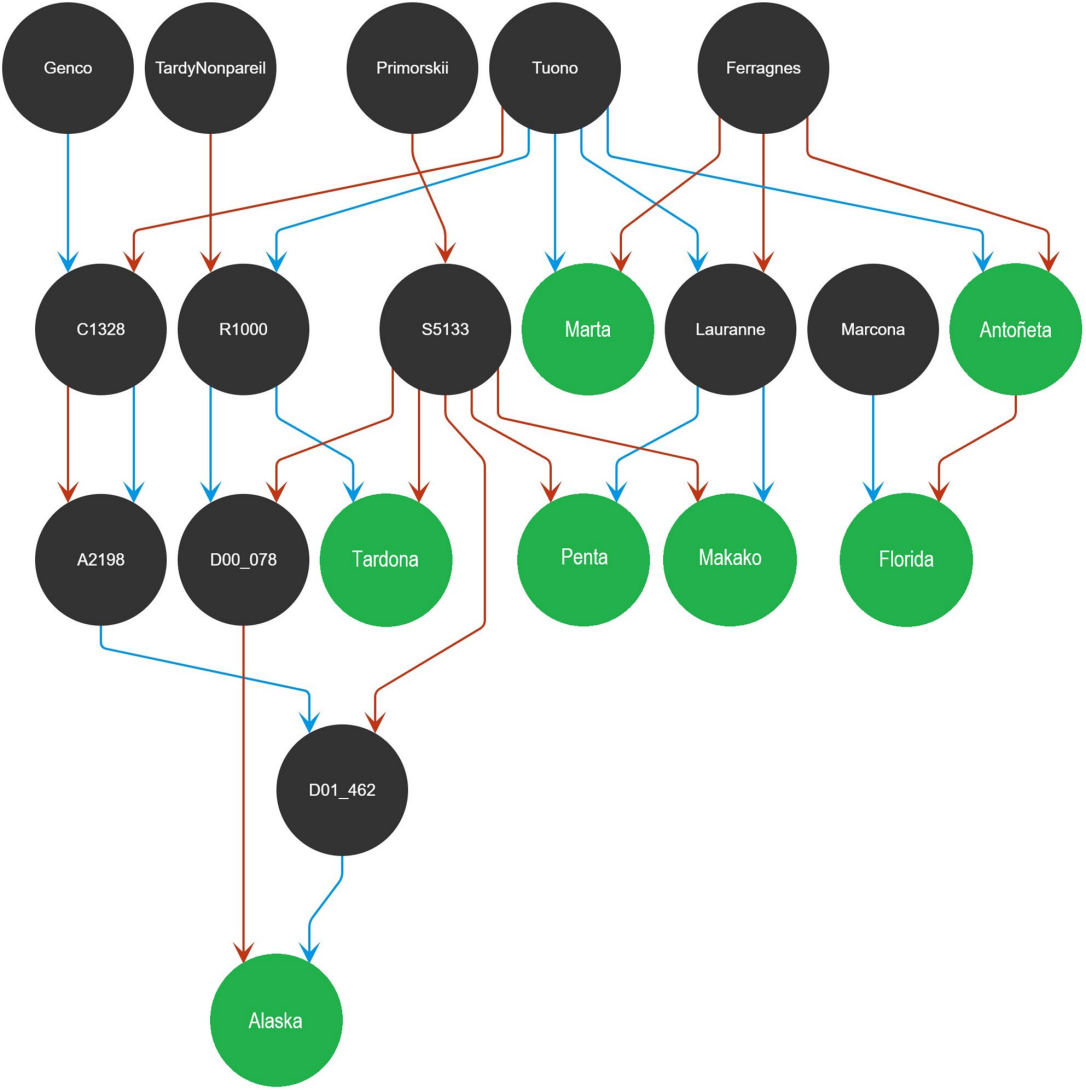
Pedigree diagram of the CEBAS-CSIC cultivars ‘Antoñeta’, ‘Marta’, ‘Penta’, ‘Makako’, ‘Tardona’, ‘Alaska’ and ‘Florida’ (shown in green in the diagram). Arrows represent parental contributions: red for female parents (dams) and blue for male parents (sires)

For this study, the first complete pedigree of the CC-ABP was created, presenting a total of 17,581 individuals. The pedigree of the CC-ABP released cultivars is illustrated in Fig. 3. After the obtaining of the pedigree, and according to the number of descendants (and relatives) of each individual, the results showed a reduced number of 38 individuals which could be considered as the “main” founders of the CC-ABP. The main contributor to this breeding program is the cultivar ‘Tuono’, having the largest number of descendants, with a total of 13,899. This pedigree was composed of 432 families, with 36 families having more than 100 descendants. Half-sib families were defined as the progenies with only one common parent, whereas full-sib families included individuals with the same two parents. There were 420 full-sib families and 12 half-sib families. For the full sib families, ‘R1000’ x ‘Desmayo Largueta’ was the largest family with 633 descendants. Among the half-sib families, the largest family, with 80 descendants, had the cultivar ‘Tardona’ as mother tree. As a result of the cross between ‘Tuono’ and ‘Ferragnès’, ‘Antoñeta’ and ‘Marta’ were obtained. ‘Tuono’ was also the parent of ‘Lauranne’ and ‘R1000’. ‘Lauranne’ progeny included ‘Penta’ and ‘Makako’, while ‘R1000’ was the parent of ‘Tardona’. Of the CC-ABP cultivars, Alaska showed the most complex pedigree, coming from two genotypes of the breeding program: D00_078, sibling of the cultivar ‘Tardona’ and D01_462, descendant of the selections S5133 and A2198 (Fig. 3).

The inbreeding coefficient of the breeding population was low, with an average of 0.10, and 68% of the individuals with an inbreeding coefficient below average. The range of coefficients was from 0 to 0.75 and the highest value was presented by four families obtained by self-pollination of selections A1180, A1340, A1342 and A1353.

### Estimation of heritability and genetic parameters

Heritability estimates obtained from both classical and mixed models are summarized in Table 3. Estimates based on between/within family variance components ranged from 0.19 to 0.76. Narrow-sense heritability, estimated via midparent-offspring regression, ranged from 0.15 to 1.05, and was generally higher than the corresponding broad-sense estimates, except for the maturity time and productivity traits, where the opposite pattern was observed. Within the Bayesian linear mixed model framework, maturity time showed the highest narrow-sense heritability (0.42), while productivity showed the lowest (0.13).

**Table 3:** Narrow- and broad-sense heritability estimates from classical methods (Midparent-offspring regression and between/within family variance components) and Bayesian mixed models

	Midparent-offspring regression	Between/within family variance components	Bayesian mixed model
	h^2^	H^2^	h^2^
Initial flowering time	1.05	0.71	0.24
Full flowering time	0.93	0.66	0.32
Final flowering time	0.79	0.76	0.14
Maturity time	0.44	0.61	0.42
Flower density	0.32	0.19	0.16
Productivity	0.15	0.40	0.13

All genetic parameter estimates within the Bayesian framework for the six traits evaluated, including repeatability, are shown in Table 4. Posterior density and trace plots were examined, revealing in general symmetrical and non-aberrant curves, confirming the unimodality of posterior densities (Figure S1-S12). As previously mentioned, the trait with highest narrow-sense heritability was maturity time (0.42). In general, a high precision (smaller intervals indicate higher degree of certainty) of heritability was observed for all traits, with a difference of ~ 0.2 between 2.5% HPD and 95% HPD. The most accurate heritability was obtained for flower density and productivity, with a 95% HPD interval between 0.12 (min) and 0.24 (max) and 0.09 (min) and 0.21 (max), respectively. Regarding repeatability, the trait with the highest value was maturity time with 0.56 and the lowest value was final flowering time with 0.15. Similarly, a high precision of repeatability was observed for all traits, with a maximum difference of ~ 0.3 between 2.5% HPD and 95% HPD in initial flowering time.

**Table 4:** Summary of the posterior modes of variance components, heritability, repeatability, and the corresponding 95% highest posterior density (HPD) intervals for the evaluated traits. The proportion of variance explained by each effect is indicated in parentheses

	Posterior mode	HPD 2.5	HPD 97.5
**Initial flowering time**			
Heritability	0.24	0.10	0.39
Repeatability	0.27	0.11	0.45
Breeding value variance	18.85 (35.40%)	16.42	20.76
Permanent effect variance	3.37 (6.33%)	2.35	4.22
Year variance	24.65 (46.28%)	14.06	129.24
Age variance	5.27 (9.90%)	1.58	22.56
Plot variance	0.11 (0.20%)	0.00	4.11
Residual variance	1.00 (1.88%)	1.00	1.00
**Full floweng time**			
Heritability	0.32	0.23	0.39
Repeatability	0.39	0.29	0.48
Breeding value variance	36.88 (35.38%)	32.78	39.52
Permanent effect variance	8.17 (7.84%)	6.54	9.75
Year variance	42.01 (40.30%)	24.55	76.64
Age variance	12.87 (12.35%)	8.29	24.23
Plot variance	3.30 (3.16%)	1.44	15.12
Residual variance	1.00 (0.96%)	1.00	1.00
**Final flowering time**			
Heritability	0.14	0.04	0.22
Repeatability	0.15	0.04	0.25
Breeding value variance	14.97 (19.10%)	13.05	16.63
Permanent effect variance	2.06 (2.63%)	1.24	2.76
Year variance	35.67 (45.50%)	15.92	209.04
Age variance	23.99 (30.61%)	8.73	97.84
Plot variance	0.70 (0.89%)	0.00	12.03
Residual variance	1.00 (1.28%)	1.00	1.00
**Maturity time**			
Heritability	0.42	0.27	0.49
Repeatability	0.56	0.38	0.65
Breeding value variance	77.31 (45.12%)	66.00	88.29
Permanent effect variance	27.08 (15.80%)	20.98	32.19
Year variance	22.74 (13.28%)	13.79	50.19
Age variance	21.02 (12.27%)	12.49	39.97
Plot variance	22.17 (12.94%)	5.31	103.83
Residual variance	1.00 (0.58%)	1.00	1.00
**Flower density**			
Heritability	0.16	0.12	0.24
Repeatability	0.28	0.20	0.34
Breeding value variance	0.85 (19.77%)	0.70	1.05
Permanent effect variance	0.44 (10.23%)	0.37	0.57
Year variance	0.37 (8.60%)	0.22	0.73
Age variance	0.89 (20.70%)	0.45	1.61
Plot variance	0.75 (17.44%)	0.30	2.45
Residual variance	1.00 (23.25%)	1.00	1.00
**Productivity**			
Heritability	0.13	0.09	0.21
Repeatability	0.25	0.16	0.31
Breeding value variance	0.67 (16.67%)	0.53	0.93
Permanent effect variance	0.40 (9.95%)	0.28	0.52
Year variance	0.61 (15.17%)	0.34	1.16
Age variance	1.00 (24.88%)	0.40	2.88
Plot variance	0.34 (8.46%)	0.08	1.41
Residual variance	1.00 (24.88%)	1.00	1.00

For genetic parameters, the permanent environmental variance component showed the most precise estimates, with the narrowest HPD intervals, across traits. The widest HPD interval for this component was for maturity time (20,98 (min) and 32,19 (max)).

Regarding the total percentage of variance explained, for all flowering time traits, the year accounted for the highest percentage, ranked from 40.30% in full flowering time to 46.28% in initial flowering time. For maturity time, the breeding values accounted for the highest percentage of the total variance explained (45.12%). In the case of productivity and flower density both residual (24.88% and 23.25%, respectively) and age (24.88% and 20.70%, respectively) accounted for the highest percentage of the total variance explained.

### Breeding values and reliability

Estimated breeding values (EBVs) and their associated reliability were calculated for each individual in the CC-ABP pedigree (Supplemental File 2). The 9,158 individuals without phenotypic data for the traits considered include the youngest trees that have not yet produced, trees that died prematurely, and trees belonging to families established for the evaluation of other traits. EBVs for these individuals were inferred solely from pedigree information. A ranking of the top 10 genotypes with the highest EBVs and its reliability for each trait was established (Table 5). This includes the 10 earliest and latest flowering individuals, relevant for breeding in warmer regions with low chilling requirements, and in colder regions where late flowering helps avoid frost damage, respectively. The ranking also highlights individuals with earlier maturity times, as well as those with the highest EBVs for flower density and productivity.

**Table 5:** Ranking of the 10 best genotypes of the CC-ABP according to their estimated breeding values for each trait, differentiated by flowering and maturity time, and by flower density and productivity

Initial flowering time				Full flowering time				Final flowering time				Maturity time				Flower density				Productivity			
EARLY																HIGH							
ID	EBV	REL	PAR	ID	EBV	REL	PAR	ID	EBV	REL	PAR	ID	EBV	REL	PAR	ID	EBV	REL	PAR	ID	EBV	REL	PAR
D99_646	-17.60	0.74		D04_408	-25.25	0.81		D99_646	-13.09	0.70		D20_409	-38.47	0.21		D98_694	2.82	0.46	yes	D98_694	1.85	0.70	yes
D99_706	-17.59	0.74		D99_706	-23.99	0.83		D99_706	-13.09	0.70		D20_382	-38.43	0.24		D01_631	2.37	0.30		D97_182	1.67	0.53	
D99_629	-15.62	0.38		D99_646	-23.61	0.82		D99_629	-12.82	0.38		D20_381	-38.41	0.25		S1233	2.26	0.56		D04_376	1.66	0.52	
D99_597	-15.61	0.43		D99_739	-23.55	0.82		D99_597	-12.81	0.44		D20_394	-38.38	0.20		D04_236	2.25	0.41		S4002	1.64	0.52	
D00_750	-15.60	0.44		D99_729	-23.52	0.81		D00_750	-12.81	0.44		D20_415	-38.38	0.21		S1219	2.22	0.58		D97_211	1.54	0.52	
D00_738	-15.60	0.41		D99_731	-23.51	0.81		D00_738	-12.80	0.41		D20_408	-38.37	0.24		S1239	2.08	0.58		S4006	1.51	0.52	
D99_626	-15.60	0.43		D99_680	-23.51	0.82		D99_626	-12.80	0.43		D20_402	-38.37	0.25		S1379	2.07	0.60		D97_175	1.51	0.48	
D99_574	-15.59	0.40		D99_677	-22.94	0.81		D99_574	-12.80	0.41		D20_426	-38.36	0.25		S1240	2.06	0.56		S5130	1.48	0.53	
D99_598	-15.59	0.38		D04_405	-22.84	0.83		D99_598	-12.79	0.39		D20_372	-38.34	0.24		S1241	2.06	0.60		S5129	1.48	0.52	
D00_760	-15.59	0.42		D22_070_S	-22.52	0.48		D00_760	-12.79	0.42		D20_405	-38.33	0.28		D00_140	2.06	0.46		S2143	1.48	0.53	
LATE																							
D08_382	31.74	0.25		D05_145	38.95	0.77		D98_672	25.50	0.66	yes												
D09_356	31.70	0.29		D09_299	30.71	0.83		D06_463	25.21	0.33													
D08_387	31.69	0.28		D09_305	30.10	0.82		D06_430	25.20	0.31													
D08_379	31.69	0.26		D10_314	29.22	0.82		D06_471	25.18	0.30													
D08_380	31.68	0.26		D10_416	28.98	0.82		D06_469	25.16	0.31													
D09_348	31.68	0.29		D10_407	28.93	0.82		D06_440	25.16	0.32													
D09_341	31.66	0.28		D08_382	28.13	0.81		D06_431	25.16	0.31													
D08_384	31.65	0.30		D10_312	28.07	0.80		D06_435	25.16	0.34													
D09_344	31.65	0.29		D10_242	27.60	0.83		D06_447	25.15	0.32													
D09_361	31.64	0.30		D10_240	27.52	0.81		D06_464	25.15	0.30													

Among all individuals in the pedigree, ‘Desmayo Largueta’ had the earliest EBVs for all flowering traits: − 21.84 for initial, − 26.54 for full, and − 17.79 for final flowering time. D99_646 was the earliest genotype from the CC-ABP for initial (–17.60) and final (–13.09) flowering time. For full flowering, the earliest EBV was observed in D04_408 (–25.25). The latest flowering genotypes were D08_382 for initial (31.74), D05_145 for full (38.95), and D98_672 for final flowering time (25.50). Regarding maturity, D20_409 had the earliest EBV (–38.47), and D10_349 the latest (64.26). The highest EBV for flower density and productivity was recorded in D98_694, being 2.82 and 1.85, respectively.

Reliability values of EBVs (Supplemental File 2) ranged from 0 to values close to 0.90 across all traits. ‘R1000’ showed the highest individual EBV reliability (0.98) for full flowering time. This trait had the highest average reliability (0.62), followed by maturity time (0.52). Final flowering time showed the lowest average reliability (0.37). For each trait, a final ranking was developed based on both EBV and reliability. According to trait-specific data structure, a threshold reliability of ≥ 0.8 was applied for full flowering time, and ≥ 0.5 for ordinal traits such as flower density and productivity. Based on these criteria, 1,196 individuals were identified as promising candidates for advancing full flowering and maturity time (i.e., EBV < 0). For example, D00_714 had an EBV of − 21.98 for full flowering time, and D17_235 had an EBV of − 32.10 for maturity time. A similar filtering strategy was used to select individuals with high flower density and productivity, yielding 169 candidates. Among them, selections S1363 and S2249 showed the highest EBV for flower density (1.65) and productivity (0.77) respectively (Supplemental File 3).

**Fig. 4:**
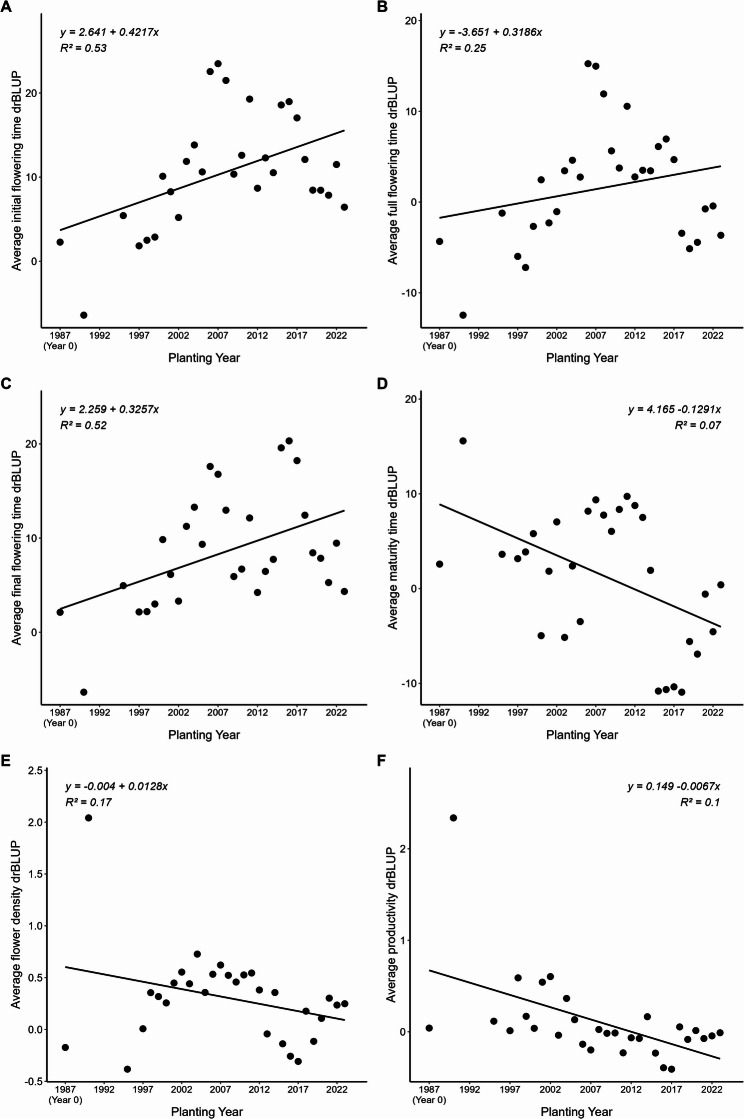
Genetic trend of average deregressed BLUPs for each trait by year of plantation. **A** Initial flowering time. **B** Full flowering time. **C** Final flowering time. **D** Maturity time. **E** Flower density. **F** Productivity

Pearson correlations were calculated between the average EBVs of each trait. All correlations were highly significant (*p* < 0.001). Strong positive correlations (*r* > 0.90) were observed between the three flowering time traits. Maturity time showed a moderate correlation with flower density (*r* = 0.30) and a lower correlation with final flowering time (*r* = 0.14). Correlations between flower density and the flowering time traits ranged from 0.25 to 0.30. Productivity showed stronger negative correlations (*r* ≈ -0.40) with all flowering date traits. Correlation between productivity and flower density was moderate (*r* = 0.49). Regression analyses revealed a significant temporal trend in drBLUPs for initial flowering time (*p <* 0.001, R² = 0.53), full flowering time (*p =* 0.005, R² = 0.25), final flowering time (*p* < 0.001, R² = 0.52) and flower density (*p* = 0.025, R² = 0.17). No significant trends were detected for maturity time (*p* = 0.16) and productivity (*p* = 0.094), with low coefficients of determination (R² = 0.07 and R² = 0.10, respectively) (Fig. 4).

## Discussion

In this study, for the first time a well-defined generalized linear mixed model was developed to estimate breeding values for key traits in the CC-ABP. This was achieved using the first complete pedigree of the CC-ABP and over 30 years of historical phenotypic records. These results will help increase selection efficiency for economically important traits such as flowering, maturity time and productivity.

### Normal distribution

The normal distributions observed for all continuous traits suggested a pattern consistent with expectations under quantitative inheritance. Additionally, even traits scored on an ordinal scale, such as flower density and productivity, exhibited distributions closely resembling normality. The observed distributions for maturity time, productivity, and flower density aligned with those reported by Sánchez-Pérez et al. [18], whose progenies were included within the studied CC-ABP pedigree.

However, these observations contrasted with the bimodal distributions reported for flowering time in almond [9, 18, 33, 61, 62], which had been associated with a major gene for late flowering (Lb) linked to the ‘Tardy Nonpareil’ cultivar, progenitor of ‘R1000’.

Patterns of flowering time distribution varied across perennial fruit species. Peach populations derived from parents with contrasting chilling requirements showed multimodal flowering time distributions [63]. In contrast, populations derived from self-pollinations or from parents with similar flowering times, such as in peach [64] and pear [65], exhibited continuous, unimodal flowering time distributions. These differences likely reflected the degree of parental divergence and experimental design more than the underlying genetic architecture of the trait.

A continuous phenotypic distribution did not exclude the presence of major genes [53]. Although flowering time in our population followed an approximately normal distribution, this did not preclude the presence of the Lb allele, especially since some CC-ABP genotypes descended from the ‘Tardy Nonpareil’ cultivar. However, as Lynch & Walsh [53] discussed, major gene effects could be masked by environmental variation and low allele frequencies, especially in large populations as those used here. Moreover, the segregating polygenic background, with individuals exhibiting diverse flowering times, contributed to a continuous distribution.

### Trait correlations

Trait correlations found in this study were largely consistent with those previously reported. High correlations were observed between flowering traits, and weak to moderate correlations were found between flowering traits and flower density and maturity time. A high correlation was also observed between flower density and productivity. These findings align with prior work by Dicenta & García [66]. Interestingly, a weak negative correlation between full flowering and maturity time was observed in this study. This contrasts with previous reports of no correlation [15, 18, 67], as well as with older studies that described positive correlations in almond and in other species such as walnut and sweet cherry [66, 68, 69]. Historically, a negative correlation between flowering time and productivity was proposed [15, 62]. This assumption persisted until the work of Grasselly & Olivier [70] who used ‘Tardy Nonpareil’ in breeding crosses and found no such correlation. Similarly, Dicenta & García [66], working with a subset of the CC-ABP, also reported no correlation between full flowering time and productivity. The negative correlation observed here is consistent with the performance of late flowering cultivars in warm regions like the one where the CC-ABP was located. These cultivars may experience problems with chill accumulation and exposure to high temperatures, which can lead to fruit drop and reduced productivity. Additionally, a significant but very low positive correlation was observed between flowering time and flower density. While the high correlation among some traits would allow for the application of multi-trait BLUP models [48], this approach was not implemented here due mainly to insufficient data for these traits. Moreover, the use of models developed for continuous traits to predict categorical traits such as flower density and productivity may reduce predictive accuracy [71] and can produce model convergence issues [72].

### Pedigree

The pedigree analysis of the CC-ABP cultivars highlighted a breeding program based on the integration of broad and diverse genetic material. This program integrated traditional germplasm, such as the Spanish cultivar ‘Marcona’, alongside international cultivars originating from diverse geographic regions including France, Italy, Russia, and the United States. This broad genetic base was key to improving adaptation to Mediterranean conditions and combining important traits like yield, quality, and enhanced resistance to pathogens and environmental stresses.

Genotypes with valuable agronomic and genetic traits, such as C1328 (‘Tuono’ × ‘Genco’), S5133 (open-pollinated from ‘Primorskii’), A2198 (selfed from C1328), D00_078 (S5133 × ‘R1000’) and D01_462 (S5133 × A2198) have been obtained and employed as parents within the CC-ABP. These genotypes have played a crucial role as progenitors in the development of improved almond cultivars, such as ‘Alaska’ originates from the cross between D00_078 and D01_462, which themselves derive from founder cultivars ‘Genco’, ‘Tardy Nonpareil’, and ‘Primorskii’. Along with ‘Florida’, they represent the first two soft-shell, American-type cultivars developed by the CC-ABP. Integrating diverse germplasm and advanced genotypes as parents has driven genetic gains for some traits in the CC-ABP, yet continued introduction of novel diversity is vital to sustain breeding progress and adaptability.

### Heritability

Previous studies have reported heritability estimates and described inheritance patterns for flowering time, maturity time, flower density and productivity in almond (*Prunus dulcis*) using traditional methods [3, 14, 15, 61, 73]. These studies generally indicated moderate to high heritability for these traits. In the present work, estimates obtained through classical approaches, midparent–offspring regression and between/within family variance partitioning, were largely consistent with those earlier findings, particularly for flowering-related traits. However, it is essential to interpret heritability estimates in the context of their underlying assumptions and limitations. Heritability, whether in the broad sense (H^2^) or narrow sense (h^2^), is a population-specific and environment-specific parameter; it does not represent an intrinsic property of a trait [52]. Consequently, heritability estimates obtained from a single environment, both in this work and in most classical studies based on traditional designs, describe the proportion of genetic variance expressed under those specific conditions and may differ if evaluated across multiple environments. In this context, the mixed-model framework used here allows a more explicit separation of genetic and environmental sources of variation than classical approaches, although genotype-by-environment (G × E) interactions cannot be fully accounted for. High heritability indicates that, within the studied population and conditions, a large proportion of phenotypic variance is attributable to genetic differences, not that the trait is genetically “determined” or unaffected by the environment. This distinction is often misunderstood, leading to overinterpretation of heritability values [74]. Moreover, classical methods can overestimate h^2^ in the presence of non-additive genetic effects (dominance, epistasis) or shared environmental effects within families. The assumption that genetic effects are purely additive rarely holds for complex traits, especially in perennial species where long generation times and clonal propagation allow accumulation of epistatic interactions. The linear mixed models implemented in this work focused on additive genetic effects. Non-additive effects, including dominance and epistasis, can contribute to phenotypic variation, but their explicit estimation requires specific experimental designs, such as clonal replication or highly structured and balanced mating schemes. In a large breeding population such as the CC-ABP, with highly unbalanced data and irregular repeated measures, separating additive from non-additive variance becomes statistically and computationally challenging. Incorporating non-additive relationship matrices would substantially increase model complexity and computational cost, often with limited power to reliably partition variance in the absence of clonal replication. Selective mating can also bias heritability estimates. When phenotypically similar individuals are crossed, both the parent-offspring covariance and the variance of the parental mean increase. While the regression slope remains unaffected under purely additive effects, non-additive genetic components can inflate this slope, leading to overestimated h² values [53]. This bias becomes evident when comparing methodologies. For flowering time traits, narrow-sense heritability was substantially higher via midparent-offspring regression, in some cases exceeding 1.0 (e.g., initial flowering time), which is biologically unrealistic. These inflated estimates underscore limitations inherent to classical regression methods, reflecting the incomplete partitioning of additive, non-additive, and shared environmental variance. The observed reduction in additive genetic variance for flowering time may be partially attributed to the recurrent crossing between genotypes derived from a limited set of late-flowering cultivars. While this breeding strategy effectively maintains the late-flowering phenotype, it potentially constrains the genetic diversity underlying this trait, thereby limiting the additive genetic variance and the response to selection. This scenario does not necessarily imply complete allelic fixation but suggests a restricted allelic diversity for flowering time within the population. Bayesian approach effectively captures this by incorporating the full relationship matrix, while midparent–offspring regression, relying on mean phenotypes of parents and offspring, is sensitive to transgenerational environmental effects and can overestimate h². In contrast, traits not subject to the same selection intensity, such as maturity time, show consistent h² estimates across methods, supporting this interpretation. For maturity time, the difference between broad- and narrow-sense heritability may reflect the contribution of non-additive genetic effects, such as dominance and epistasis, indicating that factors beyond additive variance could play a role in the inheritance of this trait. In addition, Bayesian linear mixed models, especially those using weakly informative priors, introduce mild regularization that prevents overestimation when data are limited or noisy [75, 76]. This yields more stable and conservative estimates across parameters, including heritability.

In other *Prunus* species mixed models have been used [77–81]. These studies reinforce the notion that while heritability estimates remain a cornerstone of quantitative genetics, their magnitude reflects both genetic architecture and experimental context. Without comprehensive modelling of genetic and environmental variance, including non-additive and G×E components, heritability estimates may misrepresent the true potential for selection response. In *Prunus avium*, Piaskowski et al. [82] used genomic-enabled mixed models to estimate heritability for several industry-relevant traits, reporting broad-sense heritability (H²) values of 0.83 for maturity date, 0.77 for fruit firmness, and 0.76 for fruit weight. Notably, these traits are known to be highly influenced by environmental conditions, yet the high heritability values observed highlight the strength of the genetic signal captured in the studied population. The authors also found that non-additive components played a substantial role: epistatic variance accounted for more than 40% of the total genetic variance for maturity date, firmness, and disease response, while dominance variance contributed 34% and 27% of the genetic variance for fruit weight and size, respectively. These results emphasize the importance of using comprehensive genetic models that account for non-additive variance, particularly in clonally propagated perennial species where such effects are more likely to accumulate. Furthermore, they demonstrated that high heritability estimates in complex traits must be interpreted with care, as they may reflect both genuine genetic determinism and methodological factors such as model structure, population composition, and environmental homogeneity.

### Estimated breeding values

This study highlights the value of leveraging historical phenotypic records in perennial crop breeding, especially when combined with a corrected pedigree [83]. Pedigree-based analysis, long used in animal and plant breeding [41, 84, 85], enables the monitoring of genetic relationships and inbreeding levels. The current analysis of the CC-ABP showed a higher mean inbreeding coefficient than in previous pedigree studies in almond [86], mainly due to the repeated use of a few founder genotypes [44], such as ‘Tuono’. Although pedigree-based methods were widely applied in other tree crops such as walnut [47], peach [87, 88], apple [89, 90], blueberry [91] or mango [92], this is the first known report applying this methodology to almond. In classical breeding, parental selection is result of the sum of several beneficial traits that satisfy the “breeder’s eye”, in our case the EBV-based rankings generated in this study with only few, but maybe most important, traits revealed a significant gap between genetic merit and actual parental use. For instance, high-EBV individuals were rarely used in past crosses. Traditional cultivars like ‘Ferragnès’ and ‘R1000’, often selected for late flowering, ranked relatively low in breeding value, highlighting a mismatch between selection goals and actual genetic potential.

When applying reliability thresholds, only a small proportion of the population met selection criteria for early flowering, early maturity, high flower density and high productivity simultaneously. This emphasizes the need to improve parental selection strategies in future almond breeding. Reliability thresholds were differentiated to account for the specific genetic architecture and data structure of each evaluated trait. For the continuous traits, a higher threshold was applied, leveraging the high pedigree connectivity and extensive historical records available, which ensured high prediction accuracy. The lower threshold for flower density and productivity is technically justified by their recording on an ordinal scale (1–5), which inherently increases residual variance and limits the maximum achievable reliability compared to continuous variables. As noted by Gorjanc et al. [45], in populations under selection, applying excessively high reliability thresholds for traits with limited data or elevated residual variance can reduce selection intensity and introduce bias toward over-represented families with older pedigree information, thereby limiting realized genetic gain. The thresholds were selected to balance selection intensity and genetic diversity while ensuring that EBVs provided substantially greater accuracy than phenotypic selection. One of the main limitations of this study is the lack of multi-environment data to properly estimate genotype-by-environment (G × E) interactions [53]. The use of a single experimental site limits the ability to separate environmental and genetic variance components. G × E interactions are not represented, which may influence the magnitude of the estimated variance components, potentially resulting in either over- or underestimation of genetic variance depending on the underlying genetic architecture and environmental variability. Consequently, these EBVs should be interpreted with caution when applied to other growing environments. Proper evaluation of G × E effects requires replicated genotypes across diverse environments, as demonstrated in peach [93], apple [94], and other crops using clonal trials [95–100]. In almond, these kinds of populations are not available yet, something that should be considered in the future by the almond breeding community.

Genetic trends after six generations of selection (1985–2024) reflect the historical evolution of the CC-ABP breeding objectives (Fig. 4).

The slow genetic gain observed in full flowering time was a consequence of the whole analysis of the population (early and late families together), although for each objective, early and late flowering were improved. A shift toward late flowering cultivars was promoted around 2000 to reduce frost risk, which allowed to obtain some extremely late cultivars as ‘Tardona’. More recently, a new shift in the breeding goals have occurred to obtain early flowering cultivars to meet lower chilling requirements, as climate change adaptation strategy.

Maturity time and productivity were the only traits showing a slightly negative trend, and the relatively low R² values together with the limited biological relevance suggest that the results should be interpreted cautiously. In the case of maturity time, is not a negative improvement, indeed confirm the change of direction in this breeding goal around 2012, reflecting the growing preference for earlier maturing cultivars. This kind of breeding changes have been recently observed in other breeding programs [47].

For productivity, this slightly negative genetic gain seems to start in 2000, following this tendency over 20 years (from 2000 to 2020) in CC-ABP. This decline may be partially due to the high chilling requirements of extra late-flowering genotypes that are not fully satisfied at the CC-ABP experimental field, and the high temperatures during their flowering, causing bud drop and poor fruit set. However, a more recent recovery phase appears to have occurred after 2020, where new individuals exhibiting higher breeding value (better performance). The slightly positive trend of flower density showed the lack of negative effect of extra-late flowering on this trait.

Despite that, the CC-ABP has released seven high-performing cultivars and commercialized several million of plants all around the world. These findings underscore the slow progress of conventional breeding in woody species. In future perspectives, the integration of genomic tools such as the Axiom™ 60 K SNP array for almond [36], combined with high-throughput phenotyping technologies, will allow the incorporation of molecular information to refine pedigree data and construct genomic relationship matrices. This will facilitate the calculation of genomic estimated breeding values (GEBVs), enabling the capture of Mendelian sampling variation and increasing the accuracy of selection. The CC-ABP includes a wide genetic diversity in flowering and maturity times, from very early to extra-late cultivars, and continues to develop crosses targeting key objectives, including self-compatibility, early maturity, adaptation to climate change, and drought resistance through rootstock development.

These resources and breeding efforts will provide a strong foundation for implementing advancing molecular breeding strategies in almond, with selected crosses being performed using individuals with the best EBVs, and validate in the field our predictions.

## Conclusions

This study highlights the advantages of using linear mixed model framework with Bayesian inference, to effectively partition genetic variance components in populations with unbalanced data such as the CC-ABP. These relatively medium-low heritability values reflect the complexity of the genetic architecture of the studied traits. This may help to explain the difficulty associated with breeding based only on phenotypic data. The selection scheme should be optimized by selecting the best parents to maximize genetic gain. Breeding values are an essential tool to enhance both the accuracy and effectiveness of parental selection in breeding programs. Moreover, reliable pedigree data are crucial for robust estimation of genetic parameters and breeding values. The incorporation of genomic data will improve the accuracy of breeding values and help correct pedigree information, facilitating the implementation of genomic selection in almond. 

## Supplementary Information

Supplementary Material 1.

Supplementary Material 2.

Supplementary Material 3.

## Data Availability

The datasets used and analyzed during the current study are available from the corresponding author on reasonable request.
