# Supplementary material for: Genetic evaluation of flowering, maturity time and productivity in almond (Prunus dulcis): heritability estimates and breeding value predictions
Source: BMC Plant Biol. 2026 Mar 4;26:660. doi: 10.1186/s12870-026-08458-1 (PMC13067441; doi:10.1186/s12870-026-08458-1)
Supplement: Supplementary file 1 — Supplementary Material 1. [file 12870_2026_8458_MOESM1_ESM.docx]

**Initial Flowering Time**

**
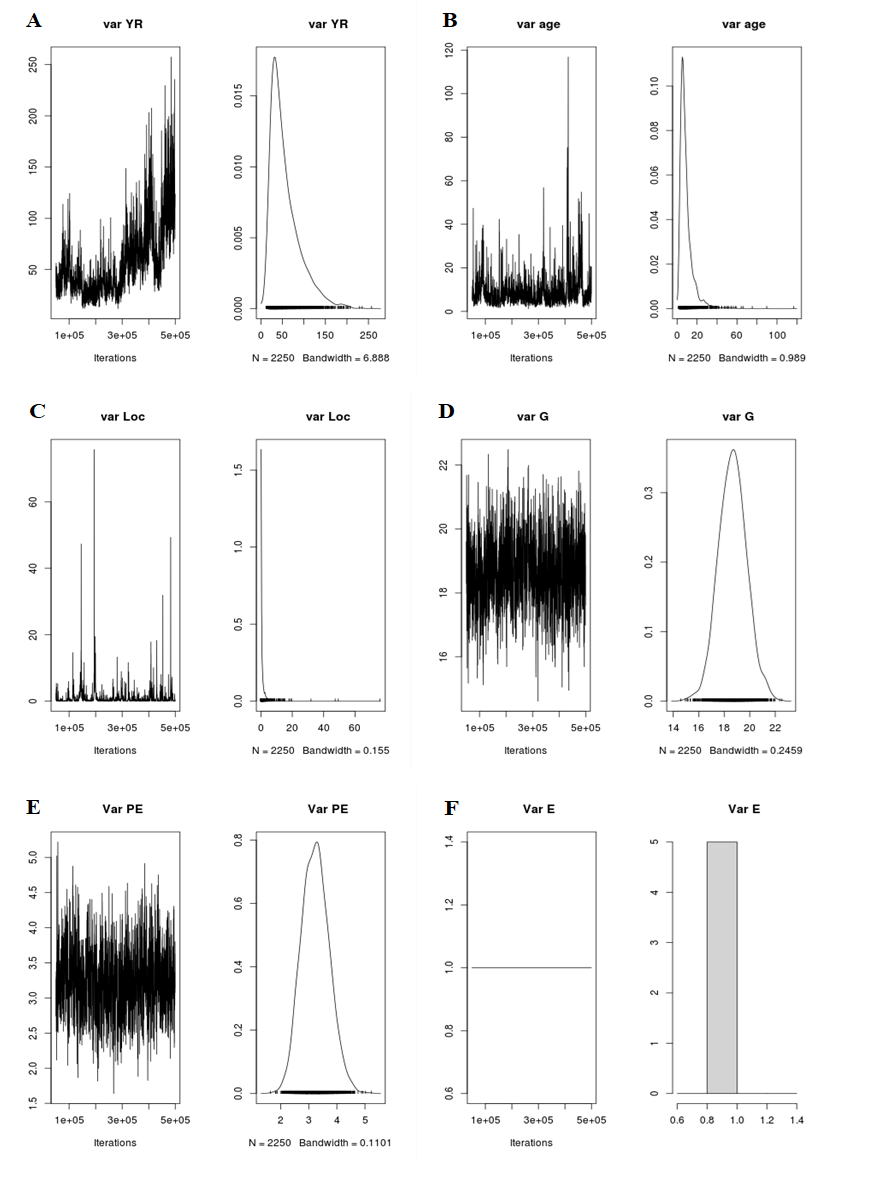
**

**Figure 1.** Markov chain trace plots and posterior density plots for the model effects of initial flowering time. **A.** Year effect (YR). **B.** Age effect (age). **C.** Parcel or location effect (Loc). **D.** Additive genetic effect (G). **E.** Permanent environmental effect. **F.** Residual effect (e). The symmetrical and non-aberrant curves indicate good chain convergence and unimodal posterior distributions.

**Figure 2.** Markov chain trace plots and posterior density plots for the heritability and repeatability parameters of the Initial Flowering Time trait.

**Full Flowering Time**

**
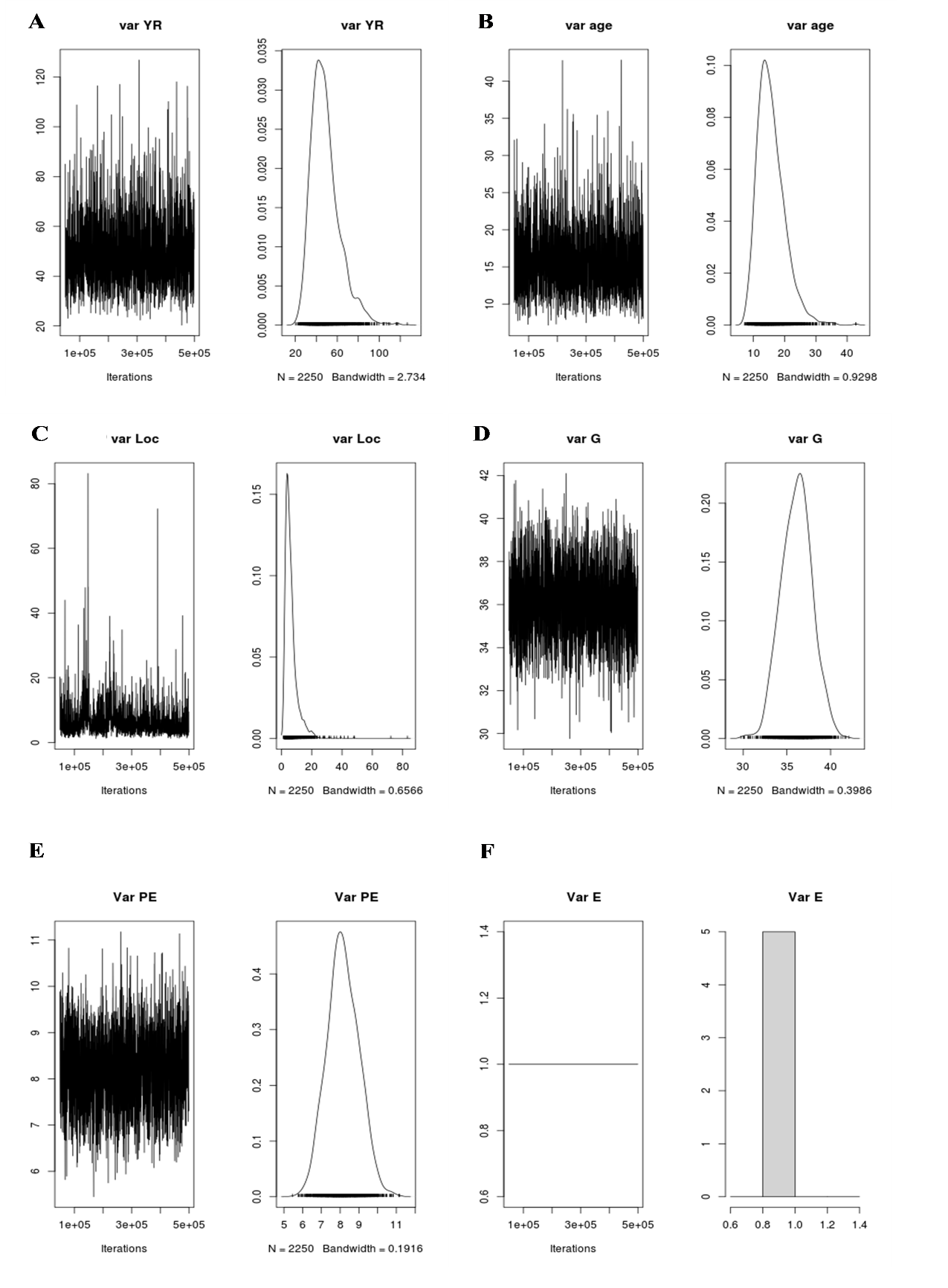
**

**Figure 3.** Markov chain trace plots and posterior density plots for the model effects of full flowering time. **A.** Year effect (YR). **B.** Age effect (age). **C.** Parcel or location effect (Loc). **D.** Additive genetic effect (G). **E.** Permanent environmental effect. **F.** Residual effect (e). The symmetrical and non-aberrant curves indicate good chain convergence and unimodal posterior distributions.

**Figure 4.** Markov chain trace plots and posterior density plots for the heritability and repeatability parameters of full flowering time. The symmetrical and non-aberrant curves indicate good chain convergence and unimodal posterior distributions.

**Final Flowering Time**

**
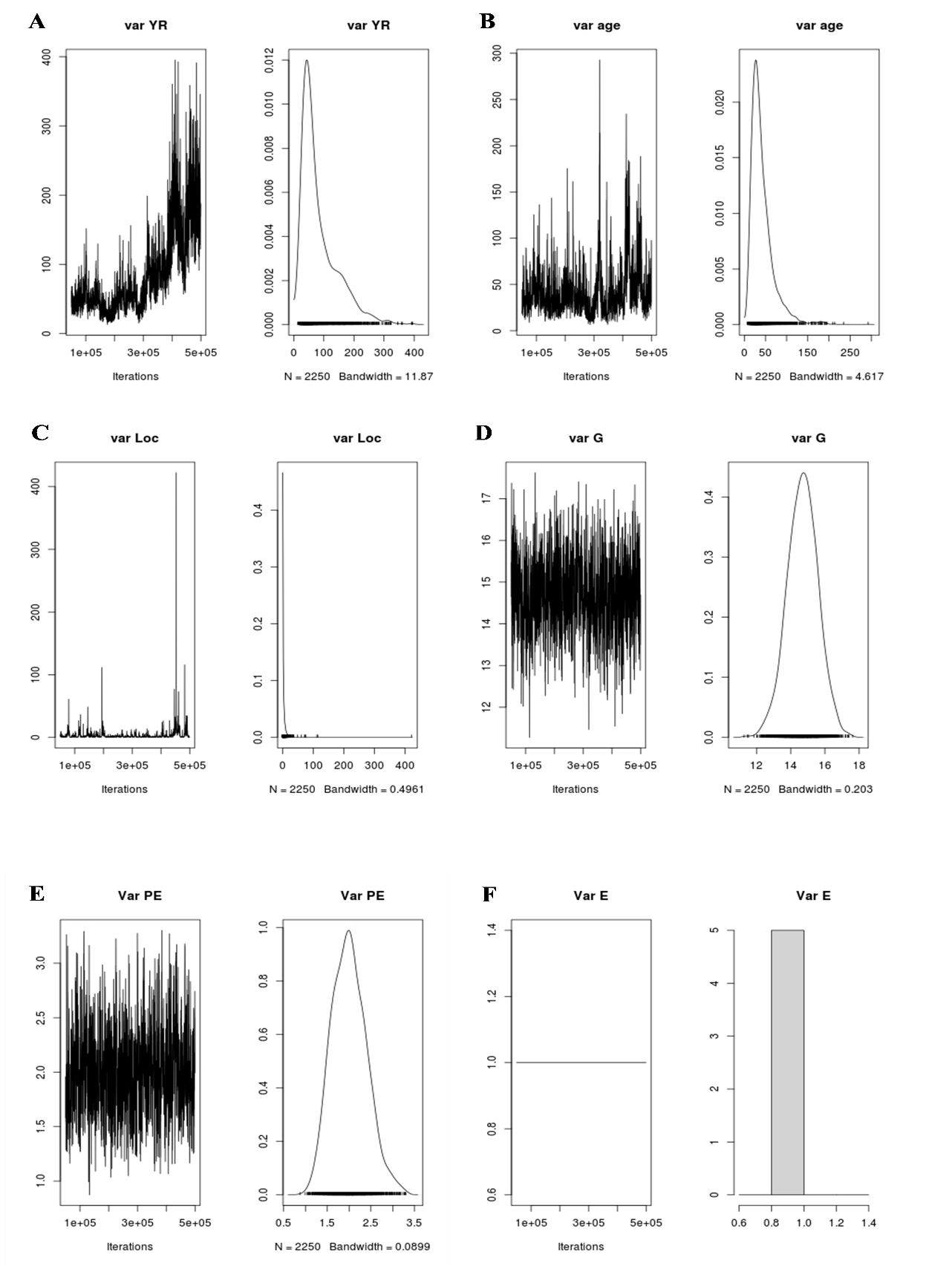
**

**Figure 5.** Markov chain trace plots and posterior density plots for the model effects of final flowering time. **A.** Year effect (YEAR). **B.** Age effect (age). **C.** Parcel or location effect (Loc). **D.** Additive genetic effect (G). **E.** Permanent environmental effect. **F.** Residual effect (e). The symmetrical and non-aberrant curves indicate good chain convergence and unimodal posterior distributions.

**Figure 6.** Markov chain trace plots and posterior density plots for the heritability and repeatability parameters of final flowering time.

**Maturity Time
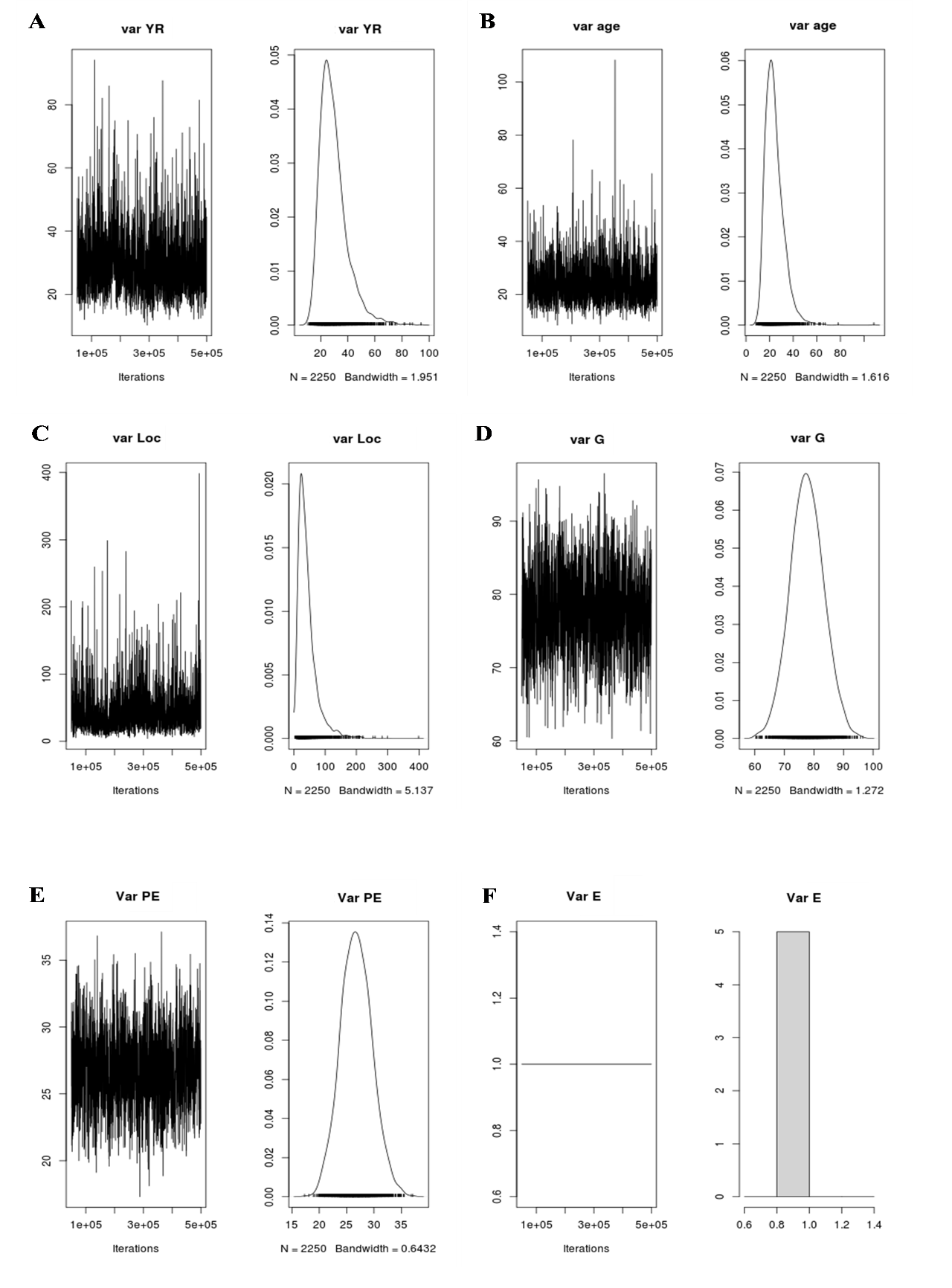
**

**Figure 7.** Markov chain trace plots and posterior density plots for the model effects of maturity time. **A.** Year effect (YR). **B.** Age effect (age). **C.** Parcel or location effect (Loc). **D.** Additive genetic effect (G). **E.** Permanent environmental effect. **F.** Residual effect (e). The symmetrical and non-aberrant curves indicate good chain convergence and unimodal posterior distributions.

**Figure 8.** Markov chain trace plots and posterior density plots for the heritability and repeatability parameters of maturity time. The symmetrical and non-aberrant curves indicate good chain convergence and unimodal posterior distributions.

**Flower Density**

**
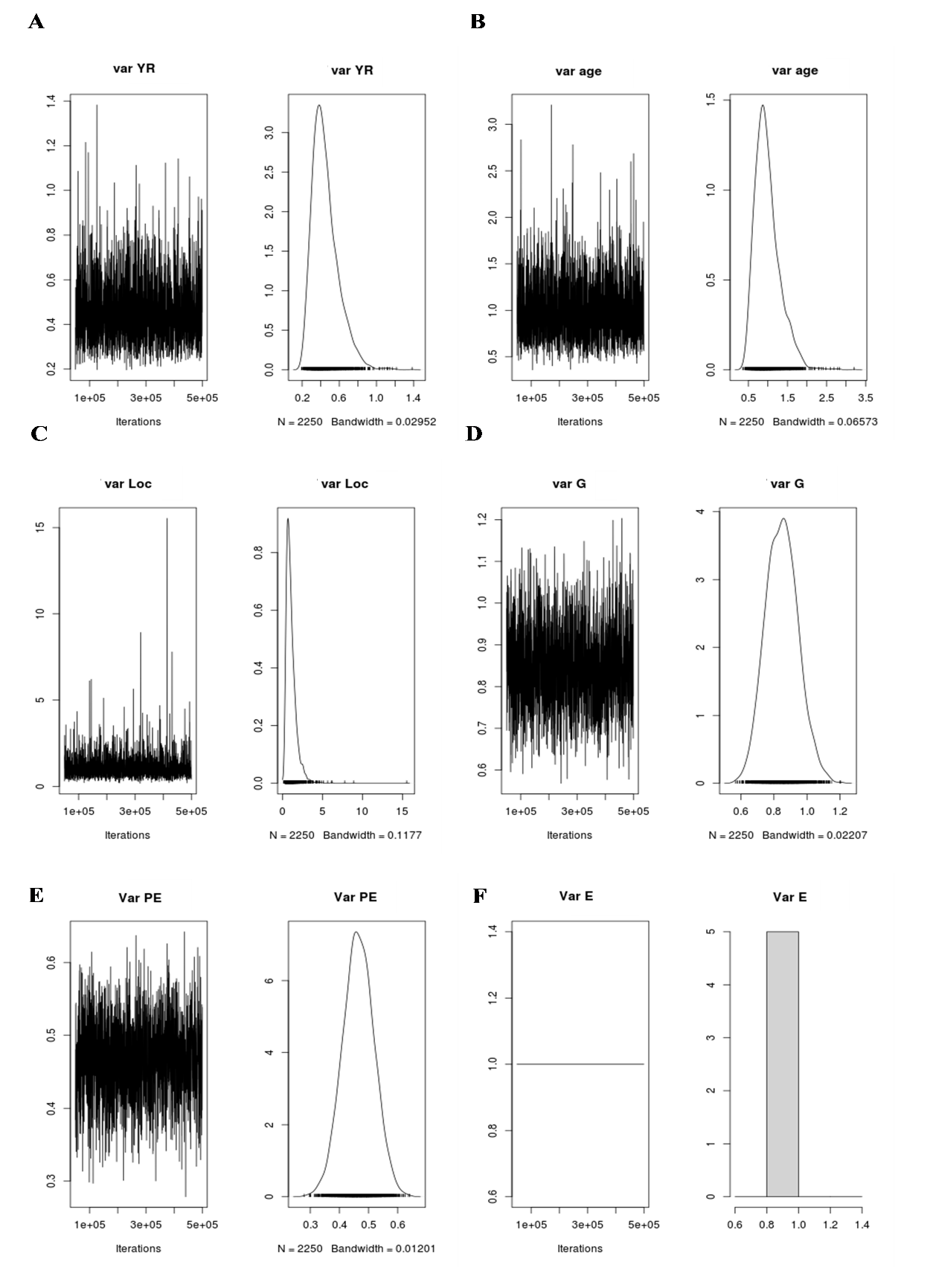
**

**Figure 9.** Markov chain trace plots and posterior density plots for the model effects of flower density. **A.** Year effect (YR). **B.** Age effect (age). **C.** Parcel or location effect (Loc). **D.** Additive genetic effect (G). **E.** Permanent environmental effect. **F.** Residual effect (e). The symmetrical and non-aberrant curves indicate good chain convergence and unimodal posterior distributions.

**Figure 10.** Markov chain trace plots and posterior density plots for the heritability and repeatability parameters of flower density. The symmetrical and non-aberrant curves indicate good chain convergence and unimodal posterior distributions.

**Productivity**


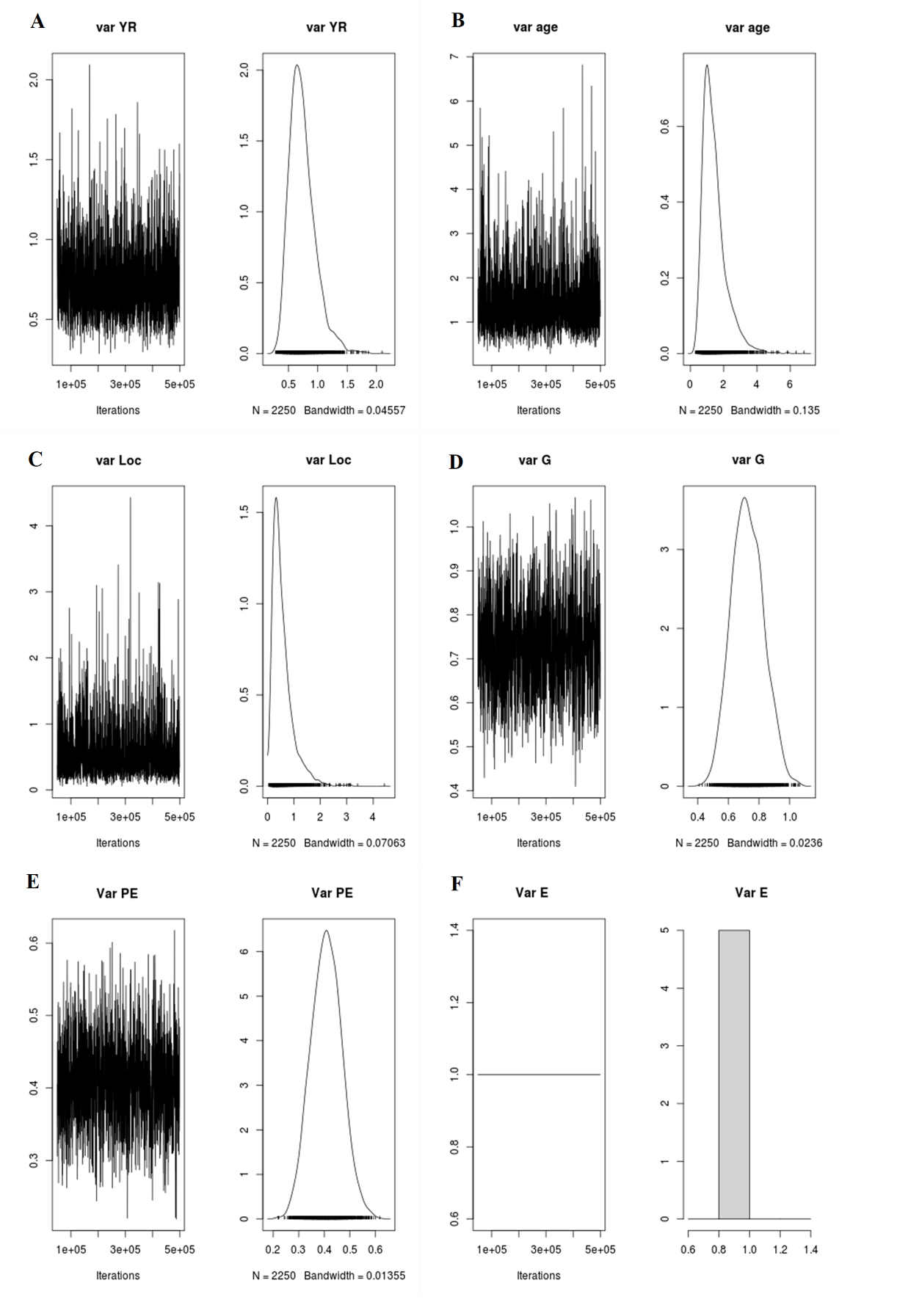


**Figure 11.** Markov chain trace plots and posterior density plots for the model effects of productivity. **A.** Year effect (YR). **B.** Age effect (age). **C.** Parcel or location effect (Loc). **D.** Additive genetic effect (G). **E.** Permanent environmental effect. **F.** Residual effect (e). The symmetrical and non-aberrant curves indicate good chain convergence and unimodal posterior distributions.

**Figure 12.** Markov chain trace plots and posterior density plots for the heritability and repeatability parameters of productivity. The symmetrical and non-aberrant curves indicate good chain convergence and unimodal posterior distributions.
